# Liquid Biopsies for Cancer: Coming to a Patient near You

**DOI:** 10.3390/jcm6010003

**Published:** 2017-01-04

**Authors:** Nithya Krishnamurthy, Emily Spencer, Ali Torkamani, Laura Nicholson

**Affiliations:** Scripps Translational Science Institute/The Scripps Research Institute, 3344 North Torrey Pines Court, Suite 300, La Jolla, CA 92037, USA; spencer.emily@scrippshealth.org (E.S.); atorkama@scripps.edu (A.T.); nicholson.laura@scrippshealth.org (L.N.)

**Keywords:** cancer surveillance, genomics, personalized medicine, liquid biopsy

## Abstract

The use of circulating tumor DNA (ctDNA) as a novel and non-invasive test for the diagnosis and surveillance of cancer is a rapidly growing area of interest, with sequencing of ctDNA acting as a potential surrogate for tissue biopsy. Circulating tumor DNA has been detected incidentally during noninvasive prenatal testing and additionally in more than 75% of known cancer patients participating in ctDNA studies evaluating its sensitivity. In the setting of mutation-based targeted tumor therapy, it shows a concordance rate >80% when compared with gold-standard tissue biopsies. Through ctDNA detection and sequencing, a simple blood test becomes a liquid biopsy for cancer, surveying a patient’s entire circulation with the goal of early detection, prognostic information, personalized therapy options, and tracking for recurrence or resistance, all with fewer or no tissue biopsies. Given the recent first-ever FDA approval of a liquid biopsy, it is important for clinicians to be aware of the rapid advancements likely to bring these tests into our practices soon. Here we review the biology, clinical implications, and recent advances in circulating tumor DNA analysis.

## 1. Introduction

DNA molecules circulating freely in human plasma, outside of cells, were first described in 1948, with practical clinical use beginning a half century later: hypothesizing that fetuses release DNA into mothers’ blood, Lo et al. demonstrated in 1997 that women who carried male fetuses had Y chromosomal DNA in their plasma [1,2]. This research transformed prenatal screening, leading to an early gestation blood test for fetal gender and chromosomal abnormalities without any intrauterine disturbance [3].

Since the rapid adoption of circulating DNA-based prenatal testing and its unanticipated detection of malignancies in a small number of pregnant women, the use of circulating cell-free DNA to diagnose cancer has been a rapidly growing area of interest [4]. This circulating tumor DNA (ctDNA) had first been described in 1977 and has since been confirmed to contain the hallmark mutations of cancerous cells [5,6]. Early studies demonstrated a quantitative correlation between the amount of ctDNA and a patient’s tumor burden [7]. Unfortunately, detection of ctDNA remained challenged by its presence in relatively low quantities, rendering it unproductive in early-stage cancer patients [8]. There are several available techniques to detect ctDNA including BEAMing, digital PCR, and next generation sequencing. Recently, however, advances in high-throughput sequencing and sophisticated computational methods as well as novel allele-specific qPCR have greatly improved the ability to detect and characterize ctDNA, with new techniques able to discover single-point mutations and track multiple genes of interest with increasing sensitivity [7,9,10,11].

Circulating tumor DNA has the potential to be a novel, non-invasive biomarker that promotes early detection at a more treatable stage, reduces the necessity of tissue biopsies, and reveals the emergence of resistance to treatment, thereby increasing the efficacy of targeted therapy. For cancers that are often detected at a late stage, including lung, pancreatic, and ovarian, a high-sensitivity ctDNA assay could function as a vastly improved screening test to detect typically terminal malignancy at an earlier, potentially curable stage. With temporal monitoring, this “liquid biopsy” shows great promise in monitoring cancer progression in real time, avoiding the significant morbidity and cost of repeat tissue biopsies.

## 2. Biology of ctDNA

The presence of cell-free DNA in the blood is well established. Fragments of DNA are constantly shed into the bloodstream during cell death, but the levels of cell-free DNA are kept relatively low due to the rapid clearance by the liver, kidney, and spleen. In general, patients with cancer have significantly higher levels of cell-free DNA as compared to healthy individuals because tumors tend to have elevated cell turnover rates and a large number of necrotic cells relative to normal tissue [12]. The median circulating plasma DNA concentration in patients with solid tumors has been noted to be three-fold higher than in healthy volunteers. Typically, dead and dying cells are cleared by filtering phagocytes, but this process does not happen efficiently for malignant cells, leading to the release of tumor DNA into the bloodstream. The rate of shedding of ctDNA into circulation is also dependent upon the location, size, and vascularity of the tumor, leading to variability in levels across patients [13]. Overall, the relative levels of ctDNA within a patient have been demonstrated to correlate with tumor burden, increasing as a tumor enlarges and decreasing with response to therapy. In colon cancer patients, for example, a tumor size of 100 g (≈3 × 10^10^ neoplastic cells) contributes to about 3.3% of the circulating DNA passed into circulation on a daily basis, whereas a much smaller proportion (<1%) can be expected for smaller, less well-vascularized tumors [14,15]. Two primary explanations for the release of ctDNA into the bloodstream have been accepted to date—passive and active mechanisms. The release of nucleic acids from necrotic cells into the bloodstream is known as the passive mechanism, with macrophages and phagocytes playing an important role in this process. Fragments of cellular nucleic acid can also be actively released, potentially as a means to condition target cellular niches at distant locations throughout the body [16].

### 2.1. Amount and Fragmentation of ctDNA

The fragmentation pattern of circulating tumor DNA also reflects tumor biology. In solid cancers, tumor necrosis creates a spectrum of DNA fragments with varied sizes, due to the random digestion by nucleases. This contrasts with apoptosis in normal tissue, which releases small, homogeneous DNA fragments. Jiang et al. used massively parallel sequencing to study the size profiles of plasma DNA samples at a single-base resolution in a genome-wide manner, demonstrating that populations of aberrantly short and long DNA molecules exist in the plasma of hepatocellular carcinoma patients [17]. Short, circulating DNA molecule abundance was elevated in the plasma of these patients relative to healthy individuals, and the relative abundance of short DNA fragments from particular genomic regions was indicative of tumor-associated copy number aberrations. Mouliere et al. demonstrated that the quantity of short, circulating DNA fragments <100 bp is directly correlated with ctDNA concentration. Optimal detection of circulating tumor DNA is obtained with amplicons <100 bp, with 98% of human colorectal cancer tumor DNA fragments being <409 bp, but with the proportion of tumor-derived DNA rapidly declining for fragments greater than 150 bp [18]. ctDNA size profiles also vary within cancer type and stage. For example, short DNA fragments are more frequent in metastatic cancers when compared with earlier stages in breast cancer [19]. Similarly, blood-based DNA integrity, defined as the relation of long to small fragments of cell-free circulating DNA, is also known to correlate with cancer progression. In a 2014 study by Leszinski et al., ctDNA analysis in the serum of patients with colorectal cancer, patients with benign gastrointestinal diseases, and healthy controls indicated that the DNA integrity index was significantly higher in patients with colorectal cancer when compared with healthy controls and with individuals with benign colorectal diseases (*p* = 0.005 and *p* = 0.006, respectively) [20]. For these reasons, circulating DNA size profiling is being examined for inclusion in a screening blood test for cancer, as it distinguishes early from late malignancies [21]. Evaluating across a diverse set of tumor types, Bettegowda et al. demonstrated that tumor stage significantly correlated with the presence of ctDNA—with 47% of Stage 1, 55% of Stage 2, 69% of Stage 3, and 82% of Stage 4 cancer patients harboring detectable levels of ctDNA.

### 2.2. Methylation Profiling

Detection of tumor-specific DNA methylation through a liquid biopsy is another feasible approach for the development of diagnostic tests for early-stage cancer. Differential methylation levels of three promoters, RASSF1A, CALCA, and EP300, in the cell-free plasma could detect ovarian cancer from healthy controls with a sensitivity of 90% and a specificity of 86.7% in a 30-patient cohort study [22]. Similarly, Lange et al. performed studies on methylated sequences in colorectal cancer, which demonstrated that methylation of the promoter region of the thrombomodulin gene (*THBD)* could differentiate colorectal cancer and control blood samples with a sensitivity of 71% and a specificity of 80% [23]. Methylated *GSTP1-*free DNA was a marker of prognosis and response to therapy in castration-resistant prostate cancer (CRPC) with detectable methylated *GSTP1* at baseline being an independent predictor of poorer overall survival and higher levels after the first cycle of chemotherapy predictive for progression measured by prostate specific antigen (PSA) [24]. Thus, methylation profiling of ctDNA provides another potential biomarker for cancer screening and surveillance. The ZNF154 CpG island is so frequently hyper-methylated in malignancy that it is being studied as a pan-cancer marker [25]. If cell-free DNA quantity, fragmentation, or methylation raises suspicion for occult malignancy, it may be further studied for ctDNA characteristics suggesting individual cancer types, as we explore below.

## 3. Diagnosis, Liquid Biopsy

The multiple-hit theory of cancer describes a series of genetic mutations—some due to exposures and many due to accumulated DNA replication errors during aging—until a combination occurs that leads to malignant cell growth. The so-called “tumor driver” hits include DNA regions that control cell division, accelerating growth promoters or blocking growth suppressors when mutated [26]. Sequencing tumor DNA provides a window into the unstable genome of the tumor itself, optimally revealing the one or more mutations contributing to unchecked growth [8]. While each tumor is therefore genetically unique, mutations in certain genes are characteristic of certain cancer types [8,27]. For example, mutated *BRAF* is seen in melanomas, *ALK* raises suspicion for lung cancer, and *EGFR* has been described in multiple cancer types including lung, colorectal, pancreatic, breast, and thyroid. A screening blood sample in the right clinical scenario and in high-risk patients could be further evaluated for genomic alterations typical of certain cancer(s), as part of the ensuing work-up to diagnose an occult malignancy.

In the opposite clinical situation, when a mass is present and tissue characterization is needed, circulating cell-free DNA can provide clues to etiology with the presence or absence of typical malignancy traits and/or driver mutations. This might be useful when more information is desired but direct biopsy is technically difficult, delayed by logistics, or inadvisable due to patient frailty. In studies pairing plasma and tumor tissue, there was >80% concordance in tumor DNA aberrations, with some results suggesting that the blood sample provided a more complete tumor profile than the tissue biopsy (i.e., ctDNA contained all or most of the tumor tissue DNA changes plus additional mutations) due to heterogeneity within primary tumors and between metastatic sites [8,28,29,30,31].This suggests that ctDNA already complements and might eventually supplant direct biopsy, with >80% sensitivity and 98%–100% specificity achieved in recent reports, and detection techniques improving rapidly [10,11,32].

### 3.1. Circulating Tumor DNA versus Tissue Biopsy

The current gold standard for clinical and investigational tumor genome profiling is paired tumor tissue/normal tissue sequencing from biopsy. Sample processing for standard, required pathological assessment can sometimes leave a tumor biopsy with insufficient material for cancer genome sequencing. Furthermore, the fraction of tumor cells relative to normal cells in each biopsy is varied, again potentially resulting in insufficient material, in turn requiring repeat aspirates or biopsies, further increasing risk to the patient [33]. Sampling of a single tumor region at the time of biopsy further limits the comprehensiveness of cancer genome sequencing due to intratumor heterogeneity. This intratumoral and intermetastatic tumor heterogeneity potentially leads to an incomplete picture of the mutational profile of the malignancy overall and may lead to the absence of information that is crucial for planning of targeted therapy regimens [29].

Circulating tumor DNA, on the other hand, provides the same key genetic information as a tissue biopsy but with some clear advantages. First, it is a mixture of DNA derived from multiple cancer sites in a single individual, providing a more representative genome of the malignancy relative to a localized biopsy. For example, Perkins et al. performed a large concordance study of ctDNA versus tissue biopsy genomic profiling in patients with advanced cancers, revealing a strong concordance between tumor biopsy data and ctDNA data, and suggesting that discordance is likely the result of a lack of sensitivity from tissue biopsies. Overall, the concordance rate between tissue biopsy and circulating ctDNA mutations was 83.3% in metastases biopsies (18 samples total) and 78.5% in primary tumor biopsies (65 specimens total). Second, the bloodstream is a readily accessible and minimally invasive source of tumor DNA, allowing repeated and longitudinal profiling of a tumor genome, for a relatively safe route to dynamic monitoring of tumor burden, heterogeneity, and response or resistance to treatment [30].

### 3.2. Circulating Tumor DNA versus Circulating Tumor Cells

Circulating tumor cells (CTCs) are another avenue for the non-invasive and dynamic profiling of cancer with many of the same benefits as ctDNA profiling [13]. However, a major hurdle in CTC analysis is a limited presence in the bloodstream. CTCs constitute as few as one cell per 1 × 10^9^ normal bloodstream cells in patients with metastatic cancer, making their detection and isolation for genomic profiling a major challenge [34]. While technology to capture and profile circulating tumor cells has advanced rapidly, the complexity and cost may limit clinical utility relative to ctDNA-based methods. Initial studies, such as that performed by Diaz et al., suggest that when both ctDNA and CTCs were present, ctDNA fragments outnumbered CTCs by 50 to 1 [8]. In a recent trial of lung cancer patients, ctDNA outperformed CTCs for detection of the *KRAS* mutation, revealing sensitivities of 96% and 52%, respectively [35].

### 3.3. Circulating Tumor DNA versus Cancer Antigens

PSA, cancer antigen (CA) 19-9, carcinoembryonic antigen (CEA), and CA-125 are protein biomarkers currently used to help detect malignancy and assess a therapeutic response in prostate, pancreatic, gastrointestinal, and ovarian cancers, respectively. However, they have performed poorly as screening assays and are proving to be unreliable for tumor prognosis and treatment response monitoring. Recently, clinical studies have demonstrated the utility of ctDNA-based biomarkers relative to protein biomarkers. For example, Diehl et al. found the quantification of ctDNA mutants and the detection of their presence/absence in colon cancer patients after surgery and chemotherapy to be more clinically useful than the cancer embryonic antigen (CEA) test [15]. In a comparison of radiographic imaging with ctDNA and CA 15-3 in metastatic breast cancer patients, CA 15-3 levels were elevated (>32.4 U per milliliter) in 71 of the 114 samples (62%), while ctDNA was detectable in 94 of the 114 samples (82%). Using a modified bootstrapping method, the study demonstrated improved sensitivity for cancer detection of ctDNA over CA 15-3: of 85% vs. 59% [36].

## 4. Personalized Cancer Treatment

Personalized cancer therapy based on a tumor’s unique genetic makeup is the crux of tumor genome sequencing and is already underway with drugs designed to interfere with the hyper-growth signals of specific driver mutations [22]. For example, trametinib in combination with dabrafenib has improved overall survival in patients with metastatic melanoma with *BRAF* V600E or V600K mutations, and erlotinib (Tarceva^®^) has significantly improved survival in *EGFR*-aberrant non-small cell lung cancer patients, even at late stages of disease [37,38]. Defining these mutations through a ctDNA liquid biopsy holds particular promise for prescribing personalized tumor therapy in cases in which tumor heterogeneity might not be fully represented with tissue biopsy or when a specimen is insufficient for all testing desired [8]. “Companion diagnostic” liquid biopsies seek to address exactly this issue, searching the ctDNA for a mutation that has a currently available targeted treatment.

### 4.1. Cancer Prognosis, Relapse, and Resistance

The bloodstream is a readily accessible and minimally invasive source of ctDNA, allowing repeated and longitudinal profiling of a tumor genome for relatively safe monitoring of tumor burden and treatment response [28]. With its half-life of less than two hours, ctDNA is dynamic and can be used to discover changes in evolving tumor genomes in real time [39]. Anticipating treatment resistance before overt clinical failure can be especially useful for patients expected to live with cancer for years. As an example of this type of prognosis, a cohort of 55 early-stage, non-metastatic breast cancer patients underwent ctDNA testing pre- and post-operatively, their tumor DNA signature representing a personalized cancer marker [40]. Positive ctDNA assays post-operatively signaled minimal residual disease, and these patients were more than four times as likely to relapse as patients with undetectable post-surgery ctDNA. Serial ctDNA testing every six months further predicted recurrence, with 93% of women who converted to ctDNA+ developing relapse compared to only 10% of women who remained ctDNA− [40].

In other studies, ctDNA-based detection preceded the clinical detection of metastasis in >80% of patients in whom ctDNA was tracked, with average lead times as high as 11 months, suggesting the potential for earlier, more targeted therapy adjustment before a decline in functional status [39,41]. Additionally, ctDNA has outperformed cancer antigens (CAs) for the detection of residual disease and recurrence. Diehl et al. found the characterization of ctDNA mutants and their presence/absence in colon cancer patients after surgery and chemotherapy to be more clinically useful than the cancer embryonic antigen (CEA) test [15]. Studies have similarly shown ctDNA to be detectible in radiologically- and biopsy-proven relapsed ovarian cancer patients whose pre-treatment positive CA-125 did not reemerge [42,43].

Mutation burden tends to increase with serial ctDNA testing and more closely matches the relapsed tumor biopsy DNA sequence than the pretreatment tumor sequence, suggesting it could be used to prescribe next-line directed therapy. *KRAS* mutations promote resistance to *EGFR*-targeted therapies, and recent studies of colorectal cancer patients demonstrated 92% sensitivity and 98% specificity of ctDNA for detecting the development of *KRAS* point mutations; *MEK1* mutation emerged in serial testing of one patient who responded to second-line treatment with the MEK inhibitor, trametinib [44,45]. Thus, ctDNA profiling has clear potential not only for the prioritization of initial therapy but also for the detection of emerging resistance and suggestion of second line therapeutic(s) [46]. Table 1 summarizes studies of ctDNA detection in common cancers.

### 4.2. Alternative Liquid Biopsy Sources

While circulating tumor DNA broadens cancer surveillance beyond a single biopsy, sampling of other body fluids potentially broadens detection even further. Central nervous system malignancies have been difficult to detect in the blood stream but are more readily detected in cerebrospinal fluid (CSF) [52]. Urine sampling adds depth and convenience: urine cell-free tumor DNA exceeds plasma sensitivity in studies of renal, bladder, and prostate cancer, but surprisingly also in some series of lung and colon cancers [53,54,55]. Additionally, tumor DNA has been detected in saliva, bronchoalveolar washings, pleural fluid, ascites, endocervical samplings, and stool [56,57,58,59,60]. From this, one can envision cell-free DNA diagnostics using the body fluid most proximate to a tumor site or even a pan-fluid screening assay, as test sensitivities continue to improve.

### 4.3. Available Liquid Biopsy

In June of 2016, Roche’s ctDNA-based detection of *EGFR* mutations in lung cancer patients was the first liquid biopsy to garner FDA approval. It is a high-specificity companion diagnostic for erlotinib, obviating the need for *EGFR* tissue testing when this blood test is positive [61]. More of these companion diagnostic liquid biopsies are being developed and will dramatically increase targeted therapy eligibility for patients too sick to undergo biopsy, too far from surgical centers, or with tumors too difficult to access safely. In the cancer screening sphere, Pathway Genomics^®^ released a white paper describing 96 common, “hotspot” tumor mutations covered by their newly available liquid biopsy, CancerIntercept™ Detect, though the FDA halted testing until validation studies are completed [62].

## 5. Limitations

The potential of a liquid biopsy in translational cancer research is clearly acknowledged and these assays have been implemented in the design of various clinical trials. However, for utilization of the liquid biopsy in a clinical setting, standardization of pre-analytical and analytical methodologies, such as blood collection, processing and storage, DNA extraction and quantification, and validation in large prospective clinical studies, is necessary. The control of different parameters in all steps—from blood drawing to ctDNA analysis—has a significant impact on the quality and accuracy of the data. The quantity of ctDNA is also a potential limitation, though new amplification technologies have begun to eliminate this concern [63]. Very low levels of mutated DNA can show as false-positive results, when the occasional DNA aberrancy does not represent a cancer clone, and as false-negative results when the level is below assay detection limits [64]. Clinical implementation of the liquid biopsy requires undertaking long-term studies with adequate sample sizes [8]. A recent evaluation into the feasibility and effectiveness of ctDNA in a large clinical study concluded that while mutation testing using plasma specimens to obtain ctDNA was attainable, it resulted in low sensitivity and a low positive predictive value [65]. The low sensitivity witnessed was most likely due to the diversity of settings in which the liquid biopsy was employed, and to the various in-house laboratory techniques used to test for the *EGFR* mutation.

## 6. Conclusions

Liquid biopsies will add a new dimension to the cancer screening and diagnosis role of the primary care physician prior to oncology referral, so it is important to understand the underlying biology and the clinical opportunity driving the rapid emergence of these tests. The minimally invasive nature of ctDNA profiling tests for malignancy without the delay, cost, and risk associated with tissue biopsy, potentially at a microscopic stage before radiologic detectability. For cancers often detected at a late stage, such as lung, pancreatic, and ovarian, a ctDNA assay could detect a typically terminal malignancy at an earlier, more treatable, even curable stage. Suboptimal sensitivities and the need to confirm the tissue of origin will limit liquid biopsy’s complete replacement of tumor biopsy for some time; however, we have discovered that “gold standard” tissue biopsies were a more limited portrait of an individual cancer than previously assumed and that concomitant liquid biopsy may add valuable, lower-morbidity treatment options for our patients.

Liquid biopsies for ctDNA have additional applications during cancer treatment, with dynamic monitoring of therapy response, early detection of resistance, and knowledge of tumor recurrence even months before clinical relapse. This could bring cancer surveillance back to the general internist’s practice, until a patient’s personalized tumor marker reemerges or evolves during serial testing. Table 2 highlights potential uses for liquid biopsy.

With the FDA’s approval of the first liquid biopsy, it is time to prepare for the clinical appearance of these new tests. Next, researchers are examining circulating RNA as a potentially improved cancer profile, and DNA methylation for its ability to signal other types of tissue damage such as the destruction of pancreatic islet cells in type 1 diabetes mellitus or oligodendrocytes in relapsing multiple sclerosis [66,67,68]. The minimally invasive nature of ctDNA profiling revolutionizes longitudinal monitoring without significant risk. The sensitivities and costs of these assays are improving at unprecedented rates with even newer technology to follow. The potential to design trials based on new driver mutations and the use of mutation-specific targeted agents across multiple cancer types should propel the use of liquid biopsy in the future. There are exciting new applications for liquid biopsy in the detection of circulating extracellular vesicles or exosomes secreted by cancer cells [69]. Continued analytical validation of ctDNA testing is key for establishing ctDNA-based assays as standard in clinical practice.

## Figures and Tables

**Table 1:** Common cancers with ctDNA detection.

Type of Cancer with ctDNA Detection	Results	References
Breast cancer	ctDNA-based detection preceded clinical detection of metastasis in 86% of patients	[47]
Breast cancer	55 non-metastatic breast cancer patients on neo-adjuvant chemotherapy; in the immediate post-operative period, 19% of available patients had detectible ctDNA, representing minimal residual disease (MRD), and 86% of these women went on to relapse during the study period	[40,48]
Colorectal cancer	metastatic colorectal cancer demonstrated 100% diagnostic sensitivity and specificity for mutant *BRAF* detection and 92% sensitivity/98% specificity for seven tested *KRAS* point mutations	[41]
Lung cancer	With tumor tissue DNA used as a reference, ctDNA demonstrated a specificity of 86% for *PI3KCA* exon 9, 88% for *EGFR* exon 19, and 100% for other measured amplicons, with an 87% (62%–96%) overall average specificity. Certain *PIK3CA* and EGFR hot-spot mutations were detected in ctDNA but not in the tissue DNA	[49]
Prostate cancer	Tumor DNA samples from the blood of 97 patients with castration-resistant prostate cancer at different times during the course of treatment with abiraterone revealed androgen receptor amplifications were present from the beginning and correlated with abiraterone resistance	[50,51]

**Table 2:** Potential uses of liquid biopsy.

Potential Uses of Liquid Biopsy
Detection of cancers in high-risk population
Monitoring for minimal residual disease
Detection of metastases before radiological evidence
Detection of response to therapy
Choice of targeted agent
Detection of new driver mutations
